# Hysteria

**Published:** 1872-09

**Authors:** 


*Hysteria :* Dr. Charcot *on Hysterical Contractions.*

In his contributions to the *Gazette des Hopitaux*, Dr. Charcot, last year, mentions that Briquet has traced with great precision the symptoms of hysterical palsy, which he calls a rare complication, not having noticed it more than six times when he wrote his book. In one case contraction occupied but one limb, in two others it put on the guise of hemiplegia, and in the last three it appeared like paraplegia. In fact, hysterical paralysis may appear in different forms, and we may verify this fact in two cases before us recently, in one of which there was the paraplegic, in the other the hemiplegic, form of the disease.

In the first case, A. E., æt. 42, had been affected with left hemiplegia for twenty months. We see that the superior limb of this side is semiflexed and considerably rigid, as is shown by the difficulty we find in increasing the flexion and the impossibility of maintaining complete extension. The left lower extremity is extended, its parts are, so to speak, in a forced attitude, the thigh strongly extended from the pelvis, and the leg from the thigh, and the foot deformed as in the most pronounced state of equinovarus; we may add that the adductor muscles of the thigh are strongly retracted. In short, all the joints are uniformly rigid, and the member in its totality forms a species of inflexible bar, which permits, when the foot is lifted, a large part of the lower part of the body of the patient to be moved. I insist on this locking of the lower limb joints because this, which is very rare indeed in cerebral apoplexy resulting from apoplectic effusions, is the rule in hysterical contraction. However, in this case before me I must notice, judging from my observations, that the permanent position of the thigh and of the leg is quite exceptional. This is an example of permanent contraction in the full acceptation of the word, and I have assured myself of this by examining the patient and finding it unmodified during the profoundest sleep, and that it gets neither better nor worse throughout the day; it is only the sleep which is caused by chloroform which makes it suddenly disappear. However, since the contraction has existed as I said for about two years, nevertheless, as you see, the nutrition of the muscles has not sensibly suffered; and I may add that the muscular contractility is nearly normal. I may notice, in passing, that when the end of the foot is strongly raised up, there is produced in the lower limb which is contracted, a convulsive tremor, which persists for a long time after that the foot being let go, the limb has resumed its former attitude. You are aware that a similar tremor is generally noticed in the paralysis with contraction due to an organic spinal lesion, *i. e.*, when the lateral tracts are *sclerosed;* I have, however, noticed it in many cases of hysterical paralysis which terminate in cure. You also see that the said affection has not the same diagnostic importance attached to it by some.

Putting aside the affection of the lower limb, all the other peculiarities might, in reality, be attributed to the effect of organic hemiplegia, which has resulted from a deep injury of the cerebellum, that is, from softening or hemorrhage. Add to this the following piece of resemblance—hemiplegia in our patient came on suddenly during an attack, after which the patient remained unconscious for several days. Whilst we notice this similarity, we see at the same time the differences, which are numerous, peremptory, and actual differences; in the majority of cases these same permit us to refer with facility the hysterical contraction to its true origin.

1st. Take notice of the want of facial paralysis, and of deviation of the tongue when put out of the mouth; you know that these phenomena always exist to a certain degree in hemiplegia of cerebral lesion with apoplectic foci.

2nd. Then note the existence of analgesia, and anæsthesia, almost absolute, and extended to the half of the paralyzed limb, face, trunk, etc. This alteration of sensation includes the skin, muscles, and perhaps the bones, and stops just at the middle line. This kind of generalized anæsthesia to one whole side of the body, head, trunk, and limbs, this limitization, I would call it geometrical, of the anæsthetized parts by a vertical plane dividing the body into equal halves, belongs to hysteria. Such phenomena are not noticed in the hemiplegia of cerebral origin, and if we had to do with spinal hemiplegia the result of lesions of one side of the medulla spinalis, the anæsthesia, as Brown-Sequard has remarked, would occupy the side of the body opposite to that of the motor paralysis.

3rd. We would also point to other distinctive characters—the patient is intelligent, and nothing makes us doubt as to the truthfulness with which she narrates the history of her attack. This is the story she tells:

She never had any hysteria before, and the disease arose at the age of 34, after a violent moral shock, with an attack of loss of consciousness; it appears that this attack had taken the epileptic form of hysteria. During the attack our patient fell into the fire, and you may remark on her face the traces of the burns made by this accident. New attacks then, clearly hysterical, supervened, and were repeated in the succeeding years, but only when she reached 40 was it that there appeared the permanent symptoms of hysteria which we are now studying; the accompanying symptoms will furnish us with characteristic traits.

*a.* Menstruation, which was regular until the moment of the first attack, became perturbed, and patient suffered from intercurrent vomiting of blood; the abdomen is considerably enlarged, and pressure on it shows a lively pain in the left ovarian region; the pain has a special character, and is accompanied with peculiar sensations, which diffuse towards the epigastric region, and which the patient says for the most part precede the attacks. These pains, as well as the swelling of the abdomen, still exist.

*b.* At the same time the patient was affected with obstinate retention of urine, which generally required the catheter.

*c.* Things were in this condition, when, in October, 1868, she had a severe attack accompanied by convulsions, and followed by an apoplectiform state, with stertorous breathing, and then, at once, hemiplegia commenced. Well, gentlemen, this notable meteorism, these pains in the ovarian region, and retention of urine, constitute a mixture of symptoms almost decisive as to diagnosis. Nothing like these is observed among the precursors of hemiplegia from cerebral causes, and, on the other hand, it is most common to see it precede the appearance of the permanent phenomena of hysteria, hemiplegia, and paraplegia. This point was not neglected by Briquet, and Laycock speaks as follows of it:

"Paralysis of the lower limbs, more or less severe, is always accompanied [he might have added *preceded*] by a corresponding degree of perturbation of the pelvic functions; the perturbation shows itself with constipation, tympanitis, and vesical palsy, with augmented or increased urinary secretion, and ovarian irritation."

*d.* When, a year ago, E. came to the Salpetriere Hospital, the hemiplegia had lasted seven or eight months. Besides the characteristically marked peculiarities noticed, the condition of the paralyzed joints might have been noticed in aid of the diagnosis of hysterical paralysis; the upper limb was flaccid, the lower limb, however, presented a very marked rigidity at the knee. This would be a considerable anomaly in hemiplegia from cerebral lesion, since in the latter the tardy rigidity is always by preference in the upper extremity.

*e.* The contraction which occupies at the moment the upper limb dates only from a few months back, and appeared after an attack. You know that the tardy contraction of hæmorrhage or cerebral softening proceeds in another way — the contraction establishes itself slowly, and in a progressive way.

Taking into account all the circumstances I have enumerated, nothing is easier than to assign to the disease of E. its true origin, and we may say the same of the following case:

B. Alb., 21 years of age, a foundling, has suffered for two years from permanent contraction of the inferior limbs, which are, as you may remark, extended and rigid. The muscular contraction, as in E., is not diminished, and the limbs are generally emaciated, partly in consequence of obstinate vomiting, which is opposed to the nutrition of the body; besides this, we notice almost complete analgesia of the paralyzed limb. Here are the circumstances which are decisive, and which permit us to be sure of the diagnosis: 1. Alb. has had hysterical attacks since the age of sixteen. 2. She has suffered from retention of urine, which frequently requires catheterism. 3. The abdomen is very tympanitic. 4. The ovarian regions are painful on pressure; if this is a little prolonged, it suddenly causes an attack. 5. The contraction of the lower limbs came on suddenly without transition, and this is a point we noted in the previous case; now you know that such like phenomena are not noticed in sclerosis of the lateral cords. As you notice, gentlemen, nothing is more simple than the clinical interpretation of these two facts, as far as diagnosis is concerned. But the prognosis may be difficult. In E. the paralysis with contraction has remained unchanged for two or three years; this contraction may give way any day, or will it persist as an incurable infirmity?

We may put these questions without giving a categorical reply. It is possible that, in spite of the long duration of the contraction, it may disappear and leave no trace behind it, and perhaps to-morrow, perhaps three days, or in some years may do nothing to invalidate this assertion; in any case, where the case take place, it may take place suddenly.

From to-day to to-morrow all may enter into order again, and if chance should grant that at this moment the hysterical diathesis should cease, the patient would take on her ordinary life. On this subject I cannot prevent myself from stopping a moment on the face of these rapid cures, which are unhoped for, of a disease which for a long time has been marked by its tenacity and its resistance to all therapeutic agents. A lively moral emotion, a combination of events which strike the imagination vividly, the return of menstruation, which has been suppressed for sometime, etc., these are the frequent occasions of such sudden cures. I have been witness to this in these cases in this hospital, which I ask your permission to narrate briefly.

1. The first occurred on a contraction of the lower limb, which had lasted at least four years. On account of the bad conduct of the patient, I was obliged to give her a vigorous lecture, and this ended by my giving her notice to quit hospital. On the next day the contraction had disappeared. This fact is the more important, because convulsive hysteria was only one of the past facts in the history of the patient; for two or three years the contraction was the sole manifestation of the grave neurosis.

2. The second case refers to a female patient also affected with contraction of one limb. True hysterical crises had disappeared for sometime. This woman was accused of theft, and the contraction which had lasted two years suddenly disappeared by the moral shock produced by the accusation.

3. In the third case the contraction had assumed the form of hemiplegia of the right side, and was stronger in the upper limb. The cure was sudden eighteen months after the commencement of the disease, after a severe contradiction she received. There was not, at that moment, any anæsthesia, and the patient, whilst she confessed having suffered curious nervous disturbance, denied the existence in past times of true hysterical fits.

It is necessary that you should know the possibility of these cases, which even in our days make it said that a miracle is present, and of which charlatans are wont to boast. In past ages such cases are frequently invoked to prove to the most sceptical the influence of the supernatural in therapeutics. In this respect you will read with interest an article published in the *Revue de Philosophie Positive* (1st April, 1869) by the respected Littre, where he alludes to a writing entitled "Un Frammento di Medicina Retrospectiva" (Miracoli di S. Luigi) in which is found the history of many cases of paralytic persons cured after making pilgrimages to S. Dionigi, at whose tomb were deposited the remains of King Louis IX. There are three cases among others, interesting to us, because of the accuracy of the particulars. They refer to women, still young, suddenly affected by contraction of one of the lower limbs, or of two of the same side, accompanied by considerable anæsthesia. In these women the cure had taken place suddenly, and in the midst of circumstances worthy of exciting the imagination; you see, gentlemen, that things have but little changed since the thirteenth century.

But if the cure of these patients is possible, or even likely, it is not necessary, and it may be that the contraction persists as an incurable affection. This is an assertion which it will be easy for me to justify; but permit me to notice first of all, that in the major part of authors you will find on this point merely vague assertions, and but little satisfactory.

I present you a woman, who is now fifty-five years of age, and who was taken eighteen years ago after an hysterical attack, with paraplegia with contraction, of which you can now recognize the principal characters. This contraction gradually or transitorily ceased or diminished. But from sixteen years ago up to this moment it has undergone not the slightest modification; we have to do, even in this case, and even yet, with a true rigidity of the muscles, with predominance of action of the extensors and of the adductors. Although the immobility of the lower limbs existed sixteen years, the ligamentous portions do not participate in the stiff joint, as far as the examination made during anæsthetic sleep has permitted us to ascertain. The deformity of the foot alone, which reminds us of varo-equinus, is not modified during chloroformization; the muscles of the leg and thigh are greatly atrophied and faradaic contraction does not exist in them. For many years past hysteria seems absent from this patient, and it is very unlikely that any event in the world can change in any way the state of her lower limbs.

What condition is then superadded to maintain the existence of the paraplegia with stiffness of the limbs? Evidently in recent cases of hysterical contraction the organic modification, whatever it may be, and whatever seat it may occupy, which produces the rigidity, is very slight and transitory, since the corresponding symptoms may disappear suddenly without transition. It is a certainty that the means of investigation which we have at present under our power, and the most diligent necropsy, *cannot* find in such cases any traces of such an alteration.

Is this the case in inveterate cases? I believe not, and feel myself authorized, founding my belief on an analogous fact, to say that in this patient there has been produced at a given moment a sclerosis of the lateral cords, a lesion which the autopsy would permit us to discover. It happened to me, in fact, once to observe, in an hysterical woman affected for sixteen years with contraction of all the limbs, which came on suddenly, a sclerosis which occupied symmetrically, and for about the whole length of the medulla, the lateral columns. Many times this woman had seen the contraction cease for a moment; but after the last attack it had been definitive. From the preceding facts it is certainly legitimate to draw some conclusions relative to the physiology and pathology of hysterical palsy.

From the considerations above cited it results that the lateral columns, or at least the posterior part of them, that which maintains the permanent contraction in cases of *sclerosis* in *plates* or in *fascicles*, these cords, I say, are marked as the seat of those organic modifications which at first were temporary, and are the causes of hysterical contraction. In time these modifications, whatever they may be, give place to more profound material alterations; and, in short, a true sclerosis takes place. Perchance this is not beyond the reach of art; but in any case it no longer permits us to hope for the sudden disappearance, which forms one of the characteristics of the disease, when not yet arrived at the more advanced phases of its evolution.

Is there perhaps a sign which permits us to indicate with certainty the character of the case, to know, for instance, if the sclerosis is or is not definitely seated in the lateral cords? I don't believe that in the actual state of science, we can point to one symptom which offers an absolute prognostic value in this point.

The convulsive shaking of the contracted limbs, whether provoked or continuous (tonic spinal epilepsy), a certain degree of emaciation of the muscular masses, a little diminution in the energy of electric contraction, ought not to be regarded, as I glean from my observations, as making us absolutely despair to see the contraction disappear without leaving any trace. On the other hand, atrophy, limited particularly to a certain group of muscles, especially when accompanied by fibrillary contractions analogous to those noticed in progressive muscular atrophy, and a notable weakening of faradaic contractility, ought to make us suppose that not only the lateral cords are profoundly affected, but that in addition the anterior cornua of the grey substance are attacked. Up to this moment I have not been able to observe these last symptoms, except in the case of old hysterical contraction, when there was no longer any hope to see the affected limb resume its normal functions.

I may add that the existence of an organic lesion of the spine more or less profound, would be almost certainly ascertained if, under the influence of sleep from chloroform, the rigidity of the limbs only slowly disappeared, or persisted in a high degree. In my opinion, until said symptoms are not well marked, it is not necessary to despair in any way. On the other hand, it is important to remember that lateral sclerosis, even when well marked, is not—and I hope to show this—an incurable disease. In the women who have attracted our attention, the contraction occupied the whole of one limb, or both, or even more.

These are, however, cases in which the spasmodic rigidity limits itself to part of a limb, to the feet, for instance, producing a kind of hysterical distortion.

Dr. R. Boddaert recently communicated such a case to the Society of Medicine of Ghent, which was very interesting; and analogous cases have been related by Little, Skey, C. Bell, and others.

If certain conveniences did not oppose themselves to it, I might refer in my turn, in all these particulars to the history of a case published by Boddaert. It suffices me to say that a young girl, æt. 22, very nervous, and belonging to a family in which nervous diseases were predominant, was attacked, three years before and suddenly, without known cause, and without having presented the characteristic symptoms of hysteria, with a painful contraction of the left leg. This contraction, which gave to the foot the typical appearance of equinovarus, had disappeared several times in the first year, but seemed to have been definitive for two years. Many muscles of the limb underwent a profound atrophy, and showed, in addition, very evident fibrillary contractions, after electric excitation. In consequence, I believe, that there is little probability of seeing the resolution of the contraction, the rather too, since it scarcely becomes relieved during anæsthetic sleep; I add the following interesting clinical note: "And this patient was free from hysterical attacks at least in the course of the last month."

### DR. BENEDICT, OF VIENNA, ON HYSTERIA.

We continue our analysis of this learned Professor's Medical teaching commenced in our May number. Dr. Benedict remarks that we must admit, above all, that hysteria may perfectly, in some cases, present the picture of what may be the result of some central or peripheral disease of the nerve-centres. There are, however, certain symptoms which almost solely are seen in hysteria, or in hysteria with its numerous complications; and there are the painful cramps of the stomach, the pharynx and larynx, and the laughing and weeping cramps; but, above all, the spinal irritation, which has become so important a symptom for electro-therapeutic treatment. To the weightiest and most constant symptoms of hysteria belong those pathological vaso-motory appearances, which exhibit themselves partly in the greater vessels and partly in the capillaries. Spasm of the vessels up to loss of pulsation take place in the great vessels, with asphyxia of the parts, coldness, pallor, pain, and anæsthesia, only it is a property of hysterical asphyxia, that it generally departs when there is real danger to the vitality of the part.

In the same way there are quasi-paralytic widening of the greater or smaller vessels, or of the capillaries, with feeling of heat and burning pain, which are quite common. A great tendency to change in the kind of symptoms forms another nearly characteristic peculiarity of hysteria. This cannot be said without some qualification, since in the case of *sclerosis in plates* a similar, though perhaps not quite so rapid, a change may be noticed. Certain symptoms are, in hysteria, at any rate, extremely uncommon—such are palsies of the facial or oculo-motor muscles; still, there can be no key to the diagnosis given from the absence of such. Hysterical palsies are frequently complicated with deep anæsthesia of the skin, muscles, and bones, so that we may, in many cases of deep motor and sensory paralysis, with great probability determine upon the hysterical nature of the case. We may also say that Basedow's (Graves') disease is a central disease of the nerve-cells; which often comes after hysteria, and is accompanied by the most marked symptoms of that disease.

We have seen, in speaking of causation, how the symptoms of hysteria commence from all of the reflected nerves of the periphery and of the hemispheres of the brain, and how they may cause alteration in distant parts of the nervous system and in other organs. In order to understand the mechanism of this reflexion, we must above all observe hysteria of the skin, etc., in their most simple forms, and especially in their connection with excitement of the cerebral hemispheres.

It is well known by all that by cerebral excitement the vaso-motor nerves of the face can be influenced in blushing and pallor, and that through similar excitation the nerves of the heart, and the sympathetic nerves of the organs of the abdomen, and many nerves of secretion may be palsied. In the same way we recognize the wide-spread cramp of the vessels, caused by psychical influences, in shuddering. Quite similarly do the cerebral hemispheres act in exciting or paralyzing on the sympathetic fibres, in the widest sense of the word, and cause thereby pathological conditions. Lasting pallor and coldness, and, on the other hand, reddening with subjective and objective rise of temperature, belong to the most common appearances remarked in hysteria of the skin, even when psychical influences are the cause of it. From this we must conclude that a far greater dependence of the sympathetic filaments exists with the activity of the cerebral hemispheres than physiologists have taught. Dr. Benedict holds that hysterical diseases of all parts are quite analogous, and arise either from spasm or paralysis of the vaso-motor nerves of the parts.

That chronic hyperæmia leads to the appearances of chronic inflammation, by means of increase of the cellular tissue and the formation of scars, is a well-known fact, and this constructs the bridge to the few pathologico-anatomical facts which we know concerning hysteria, namely, to those scleroses pointed out by Charcot, especially the strings in hopeless cases of hysterical contraction, and such cases of myelitis as that described by Benedict in 1871, occurring in hysterical paralysis. "Spasms of the vessels, with its direct consequences, and secondary consecutive atrophy, or palsy of the vessels, with or without consecutive inflammation and their consequences, are thus become, in the highest degree, the probable mechanism of hysteria, so that this disease ought soon to be withdrawn from the list of simple neuroses. The influence on the vaso-motor nerves is, as we know from *ætiological* considerations, a reflex one, and we can describe the hysteric situation as an inborn or acquired increase of the reflex irritability of the vaso-motor nerves." In no other disease is it so clear that the physician must not only be a man of science, but also a psychologist. The will has great influence over the attacks. In many cases religion does more than medicine to influence hysterical cases. Amenorrhœa is often the consequence of hysteria, and diseases of the genital organs are a frequent cause of it. Dr. Benedict speaks in great praise of Dr. Chapman's ice-bags in the treatment of hysteria.—*The Doctor.*

